# Adolescent gambling behaviour, a single latent construct and indicators of risk: findings from a national survey of New Zealand high school students

**DOI:** 10.1186/s40405-016-0017-9

**Published:** 2016-08-03

**Authors:** Fiona V. Rossen, Mathijs F. G. Lucassen, Theresa M. Fleming, Janie Sheridan, Simon J. Denny

**Affiliations:** 1 Social and Community Health, School of Population Health, Faculty of Medical and Health Sciences, University of Auckland, Private Bag 92019, Auckland, 1142 New Zealand; 2 Department of Health and Social Care, The Open University, Milton Keynes, UK; 3 Department of Psychological Medicine, University of Auckland, Auckland, New Zealand; 4 Department of Paediatrics: Child and Youth Health, University of Auckland, Auckland, New Zealand; 5 School of Pharmacy, University of Auckland, Auckland, New Zealand

## Abstract

This study explores underlying latent construct/s of gambling behaviour, and identifies indicators of “unhealthy gambling”. Data were collected from Youth’07 a nationally representative sample of New Zealand secondary school students (N = 9107). Exploratory factor analyses, item-response theory analyses, multiple indicators-multiple causes, and differential item functioning analyses were used to assess dimensionality of gambling behaviour, underlying factors, and indicators of unhealthy gambling. A single underlying continuum of gambling behaviour was identified. Gambling frequency and ‘gambling because I can’t stop’ were most strongly associated with unhealthy gambling. Gambling to ‘feel better about myself’ and to ‘forget about things’ provided the most precise discriminants of unhealthy gambling. Multivariable analyses found that school connectedness was associated with lower levels of unhealthy gambling.

## Background

Gambling has become a widely available activity in modern societies (Turchi and Derevensky 2006). Evidence suggests that it has become a popular past-time, not only for adults, but also for children and adolescents (Derevensky and Gupta 2000; Gupta and Derevensky 1998a; Jacobs 2000; Splevins et al. 2010; Turchi and Derevensky 2006). Whilst for many youth involvement in gambling does not result in problematic behaviour, other adolescents go on to experience serious issues as a result of their gambling. While the documented rates of youth involvement in gambling vary widely (perhaps due to methodological discrepancies), a recent review of studies of young people’s gambling in North America, Europe and Oceania reported that 20–90 % of young people had gambled in the past year (Volberg et al. 2010) and 0.8–13.0 % were involved in problem/pathological gambling (Volberg et al. 2010).

Certain groups of young people are at greater risk of unhealthy gambling. For instance, male youth are more likely than their female counterparts to engage in gambling (Blinn-Pike et al. 2010; Darling et al. 2006; Delfabbro et al. 2005; Huang and Boyer 2007; Raisamo et al. 2013; Turchi and Derevensky 2006), gamble more frequently (Delfabbro et al. 2005; Gupta and Derevensky 1998a, b) and do so on a wider range of activities (Delfabbro et al. 2005). The prevalence of problem gambling amongst youth from different ethnic groups has not been widely investigated (Blinn-Pike et al. 2010). Despite this lack of evidence, a few studies have indicated that indigenous youth and those from non-majority ethnic groups may be less likely to gamble than their peers. However those non-majority ethnic youth that do gamble are at greater risk of experiencing problem gambling (Delfabbro et al. 2005; Volberg et al. 2010).

The literature has highlighted that adolescent problem gamblers have an array of co-existing problems (Gupta and Derevensky 2000; Shead et al. 2010; Volberg et al. 2010), with researchers theorising that gambling is often undertaken as an attempt to manage or resolve other underlying issues (Gupta and Derevensky 2000; Ste-Marie et al. 2002). Furthermore, risk factors associated with problem gambling, such as mental health issues (e.g. depression and anxiety) may also be observed in other potentially harmful behaviours in adolescence, for instance substance misuse (Derevensky et al. 2003; Shead et al. 2010). While there has been some focus on the identification of risk factors for youth problem gambling, little is known about the protective factors that insulate young people against unhealthy gambling (Dickson et al. 2008), or the extent of their positive influence over risk factors.

There is a dearth of information on the ‘warning signs’ for problem gambling, meaning that preventive measures can be hampered. Policy makers and those working clinically in the problem gambling field would benefit from an exploration of indicators of when gambling behaviour is becoming unhealthy, and a non-dichotomised examination of youth gambling would allow for a comprehensive analysis of factors associated with unhealthy gambling. This study presents findings from research which explored youth participation in gambling and the impact of problem gambling, and identifiable risk and resiliency factors. To the best of the authors’ knowledge this is the first systematic assessment of risk and protective factors of youth gambling based on data from a nationally representative sample of young people. The objectives of this study were to: (1) explore the existence of an underlying latent construct/s of gambling behaviour—i.e. to create an underlying latent continuum of gambling behaviours from ‘less unhealthy’ to ‘more unhealthy’; (2) identify ‘red flags’, or indicators of unhealthy gambling behaviours; and, (3) explore and identify associations between gambling behaviour (as measured by the latent construct), demographic variables, and variables that were hypothesised to fulfil risk or protective factors.

## Methods

### Sample

Data for this study were collected as part of Youth’07, a nationally representative survey of the health and wellbeing of New Zealand secondary school students conducted in 2007. A brief overview of the Youth’07 survey design and methodology is included below, while a comprehensive description has been provided elsewhere (Adolescent Health Research Group 2008). A total of 9107 randomly selected secondary schools students (equating to 3.4 % of the total 2007 New Zealand secondary school roll) aged between 12 and 19 years old completed the survey. Final response rates were 84 % for schools and 74 % for students. The comprehensive 622-question survey was administered via internet tablets (hand held computers). Screening questions were used to ensure that only those who had experienced a particular issue were asked more detailed questions (e.g. those who had not smoked cigarettes were not asked further questions about smoking). Hence, students generally answered considerably fewer questions than the survey total number of questions.

### Measures

*Involvement in gambling* was assessed through a series of questions. The first question determined *past year gambling*: (“In the last year have you ever gambled or bet money on things like Lotto, Instant Kiwi, Pokies etc., or bet money with friends?”). Students who answered affirmatively were then asked five questions to determine the extent of their involvement in gambling behaviour over the past year, in particular:

- *Gambling activities* “In the last year have you gambled or bet money on any of these?” Students could select as many of the following options as appropriate—Instant Kiwi (scratchies); Lotto (including Strike and Powerball); bingo or housie; pub or club pokies (i.e. electronic gambling machines/EGMs); casino pokies or tables (e.g. roulette); TAB betting; internet (e.g. internet casinos); bets with friends; 0900 phone games; cards or coins; other; and/or, none of these.
- *Frequency of gambling* “During the last four weeks, about how often did you gamble?” Affirmative responses to this item were aggregated to ‘several times a week/most days a week,’ and the broader category ‘once a week or less’.
- *Money spent on gambling* “How much money would you usually spend each week on bets or gambling?” Responses to this item were collapsed into two categories—‘less than NZ$20’ (less than approximately US$13) and ‘NZ$20 or more’.
- *Time spent gambling* “How much time would you usually spend each day on bets or gambling?” Responses were aggregated to ‘less than 1 h a day’ and ‘1 or more hours per day’.
- *Reasons for gambling* “Why do you gamble or bet money?” Students were able to select as many reasons as appropriate from the following list: “to have fun”; “to win money”; “because I am bored”; “to relax”; “to feel better about myself”; “to forget about things”; “because my friends do”; “because my family does”; “for a challenge”; “because I can’t stop”; “because I am short of money”; “to get a buzz”; and, “none of these”.

#### Variables hypothesised to fulfil risk or protective factors

Variables selected for inclusion in this set of analyses were identified a priori by reviewing the literature and based on consultation with the study’s advisory group, which consisted of researchers and clinicians with expertise in the prevention and treatment of problem gambling.

- *Wellbeing* was assessed by the WHO-Five Wellbeing Index (Bech et al. 2003). This Index is made up of five items that use a six-point Likert scale, from 0 (at no time) to 6 (all of the time). Three underlying constructs are measured: positive mood, vitality, and general interests. Response scores were summed to provide an overall score, with higher scores indicating greater wellbeing.
- *Depression* was measured by the Reynolds Adolescent Depression Scale-Short Form (RADS-SF) (Norris 2007). This scale consists of ten items, each of which has four response options: “almost never” “hardly ever”, “sometimes”, and “most of the time” (significant depressive symptoms ≥ 28, total score = 40). The overall score provides an indication of affective status, with higher scores being an indicator of greater depressive symptomatology.
- Data on *weekly alcohol use* were gathered by asking those students who had drank alcohol at least once in their lifetime “During the past 4 weeks, about how often did you drink alcohol?”; responses to this item were aggregated to ‘weekly or more often’ and ‘less than weekly’.
- Scales were developed to provide measures of *social connectedness* in three domains: *family, friends,* and *school*. The scale for family connectedness included nine items that focused on: whether students had fun with their family; if they got along with their family; and, their relationship with their family, in particular their mother and/or father (or people that functioned as parents). The scale for connectedness to friends incorporated six items which covered various aspects of friendships, such as having caring friends and fun with friends. The school connectedness scale included eight items that enquired about students’ connectedness to their school. All three scales demonstrated adequate to good internal consistency, with Cronbach’s alphas of 0.85 (family), 0.78 (friends) and 0.60 (school).
- *Weekly income* there was no specific question about whether or not students received a weekly allowance, hence income was determined by asking students “How much money do you usually earn each week?”
- *Age*, *sex*, and *ethnicity* were self-reported.
- *Neighbourhood socio*-*economic deprivation* was measured using the New Zealand Deprivation Index (NZDep2006) (Salmond et al. 2006). NZDep2006 was also used to identify whether students resided in urban or rural settings.

### Analysis

The analyses for this study were completed in 2015 and includes only those students who had gambled in the past year from the total Youth’07 sample of 9107 students (26.7 %; n = 2234). These students were significantly (*p* ≤ 0.05) more likely to be male (31.3 % male and 21.5 % female), older (24.9 % ‘13 or less’, 23.7 % ‘14 years old’, 26.2 % ‘15 years old’, 28.5 % ‘16 years old’, 31.9 % ‘17 or older’), and from neighbourhoods with lower levels of socio-economic deprivation (28.2 % low, 27.5 % medium, 23.3 % high). No differences were observed for past-year gambling status according to ethnicity or rural/urban category.

Exploratory factor analysis was used to assess dimensionality of gambling behaviour and the number of underlying factors. The relationship between participants’ responses to each item and their level on the underlying latent gambling continuum/s were examined using two-parameter logistic item-response theory (IRT) models. Item characteristic curves (ICC) illustrate these relationships, which were characterised by item severity and discrimination parameters. Item severity provides an indication of the position of the item characteristic curve in relation to the latent continuum: higher severity values indicate that an item is associated with a higher severity of gambling behaviour (i.e. unhealthy gambling behaviour). Discrimination parameters provide an indication of the accuracy or precision of each item in distinguishing between those participants with levels of the latent continuum above, and those participants with levels below, the item’s severity. A low discrimination estimate indicates that an item is unrelated to the underlying construct or that the item is poorly defined.

Multiple indicators-multiple causes (MIMIC) analyses were used to examine the relationship between demographic variables and the underlying continuum of problem gambling behaviours and predictors of unhealthy gambling behaviour. Differential Item Functioning (DIF) by demographic variables (i.e. age, sex and ethnicity) was used to examine differences in how demographic groups respond to individual questions, independent of the underlying problem gambling continuum. The presence of DIF would indicate measurement non-equivalence or item response bias across groups.

Analysis of risk and protective factors progressed in two models. The first model examined independent associations between the latent gambling continuum and risk and protective factors, whilst accounting for student-level demographics. In model two, the association of all significant risk and protective factors and student-level demographics were examined simultaneously in relation to the latent gambling continuum. Multivariable, factor, IRT, and MIMIC analyses were undertaken using Mplus (Muthen and Muthen 1998–2007). All analyses accounted for the clustering of students within the same school and the unequal probability of selection.

### Human participant protection

Principals provided consent on behalf of their schools and students that were selected and their parents were provided with information sheets about the study. Students provided their consent on the day of participating. Ethical approval for the study was granted by the University of Auckland Human Subject Ethics Committee.

## Results

### Underlying constructs of unhealthy gambling behaviour

Exploratory factor analysis (EFA) was used to assess the dimensionality of the gambling behaviour items relating to reasons for gambling, frequency of gambling, and time and money spent gambling. The scree plot of the eigenvalues (5.301, 0.838, 0.294) and the ratio of the first to the second eigenvalue (5.301/0.838 = 6.326) suggested the presence of a single, dominant factor underlying these gambling items. This one-factor model demonstrated excellent fit indices (RMSEA = 0.047, CFI = 0.982, TLI = 0.973), and provided evidence of undimensionality of the variables, meaning that it was suitable for IRT modelling (see Table 1 for details on item factor loadings, discrimination and severity parameters).

**Table 1:** Two-parameter item-response theory model of gambling behaviour

	Prevalence (%)	Factor loadings	Item discrimination		Item severity	
			Estimate	Standard error	Estimate	Standard error
I gamble to relax	6.0	0.848	1.603	0.133	1.841	0.068
I gamble to feel better about myself	2.9	0.981	5.044	1.913	1.928	0.060
I gamble to forget about things	3.7	0.914	2.246	0.256	1.954	0.073
I gamble because I can’t stop	1.6	0.885	1.902	0.295	2.431	0.116
Gamble ‘several times a week’ or ‘most days’	2.6	0.806	1.361	0.206	2.412	0.159
Spend $20 or more per week on gambling	5.0	0.789	1.284	0.123	2.101	0.100
Spend 1 or more hours per day gambling	3.1	0.866	1.730	0.240	2.189	0.101

This factor was conceptualised as an underlying continuum of ‘unhealthy’ gambling behaviour and centres on motivations/reasons for gambling and levels of expenditure (in terms of time, frequency, and money) on gambling.

### Identifying the ‘red flags’ of unhealthy gambling behaviour

**Fig. 1:**
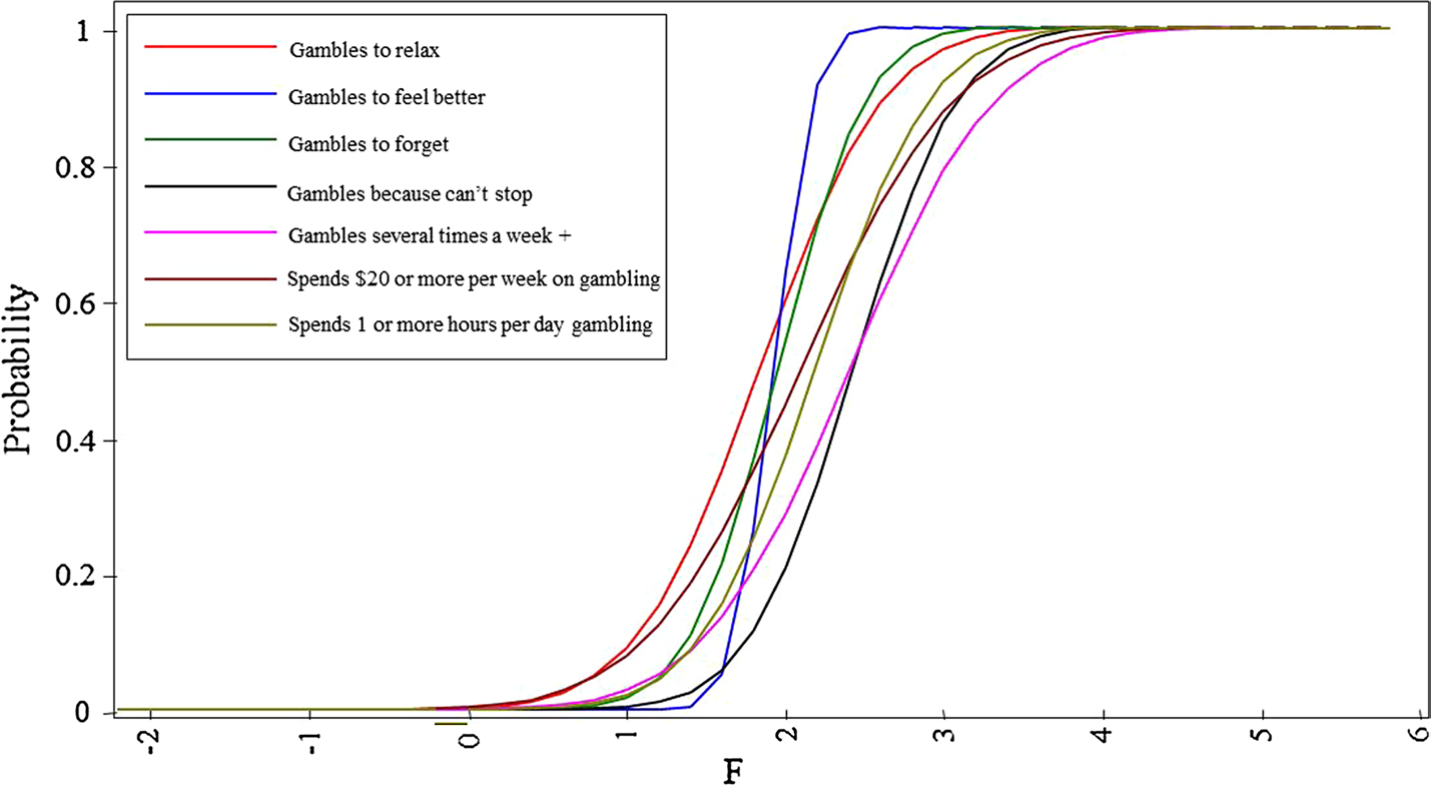
Item response curve

Figure 1 illustrates the item characteristic curves for the gambling items. All of the gambling items exhibited good to high discriminative power (range 1.284–5.044) in distinguishing behaviour along the gambling continuum, from less unhealthy to more unhealthy behaviour. Gambling “to feel better about myself” was the item with the highest discrimination (5.044, note the steepest slope in Fig. 1) and was most able to accurately discriminate students along the gambling continuum. Gambling “to forget about things”, “because I can’t stop”, and “to relax” also had high item discriminations (2.246, 1.902, and 1.603 respectively). The amount of money spent on gambling had the lowest item discrimination (1.284; note the least steep slope), meaning that it was least able to accurately discriminate students along the continuum of gambling behaviour. As gambling “to relax”, “to feel better about myself”, and “to forget about things” had the lowest item severities, these items may therefore act as early indicators that gambling behaviour is more unhealthy.

### Unhealthy gambling, depression and wellbeing

Measures of depression and wellbeing were used to determine construct validation. The underlying gambling behaviour continuum was significantly correlated with standardised measures of depression (RADS-SF) and wellbeing (WHO-Five Wellbeing Index). ‘More unhealthy’ gambling behaviour was positively associated with depression and negatively associated with wellbeing, in that students with ‘more unhealthy’ gambling behaviour had significantly higher depression scores (r = 0.162, *p* < 0.001) and significantly lower wellbeing scores (r = −0.113, *p* < 0.001) than students who were gambling at ‘less unhealthy’ levels.

### Unhealthy gambling behaviour and different demographic groups

**Fig. 2:**
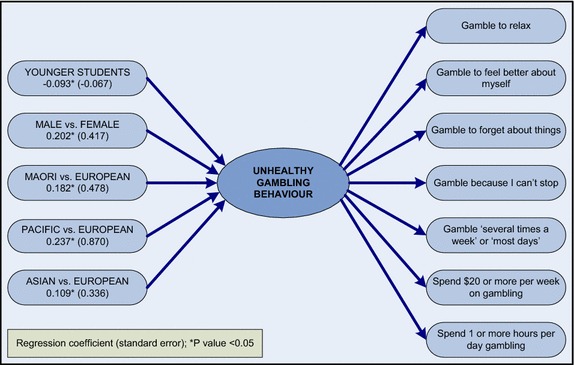
MIMIC model of unhealthy gambling behaviour. Relates to students who have gambled in the past 12 months (N = 2234)

MIMIC modelling was carried out to determine if there were significant direct effects between demographic variables (i.e. age, sex, and ethnicity) and the gambling items, and whether any of the demographic subgroups were at increased or decreased risk of unhealthy gambling behaviours (see Fig. 2). Differential Item Functioning (DIF) demonstrated item equivalence for seven gambling items (gambling “to relax”, gambling “to feel better”, gambling “to forget”, gambling “because can’t stop”, frequency of gambling, money spent gambling, and time spent gambling) across the age, sex and ethnic groups of students. The MIMIC model provided a good fit for the data (RMSEA = 0.030, CFI = 0.979, TLI = 0.973) and ‘unhealthy gambling’ behaviour was shown to vary across each of the investigated demographic variables. Higher levels of ‘unhealthy gambling’ behaviour were associated with being younger, male, and being an ethnic minority.

### Risk and protective factors for gambling behaviour

Analyses for Model One resulted in a set of variables that were significantly associated with scores on the gambling continuum (see Table 2 for estimates and standard errors) while adjusting for age, sex, ethnicity and neighbourhood socio-economic deprivation. Ten variables were associated with increased risk/higher scores on the latent gambling continuum (i.e. ‘more unhealthy’ gambling behaviour). Three domains were negatively associated with scores on the gambling continuum, i.e. they were protective with regard to unhealthy gambling behaviour: connectedness to family, connectedness to friends, and connectedness to school. As levels of connectedness in each of these domains increased, the mean score on the gambling continuum decreased.

**Table 2:** Multi-model correlations between risk and protective factors for unhealthy gambling behaviour

Item/variable associated with increased risk of unhealthy gambling	Model 1^†^ Estimate (Standard error)*	Model 2 Estimate (Standard error)*
*Demographics*		
Age (younger vs. older)	–	−0.092 (0.065)
Sex (male vs. female)	–	0.580 (0.187)*
Māori (vs. European)	–	0.227 (0.177)
Pacific (vs. European)	–	0.736 (0.282)*
Asian (vs. European)	–	0.535 (0.288)
Living in neighbourhood with higher levels of deprivation (vs. lower levels of deprivation)	–	0.044 (0.032)
*Risk factors*		
Usually earns $100 or more per week	0.387 (0.111)*	0.322 (0.150)*
Gamble because		
My friends gamble	1.436 (0.122)*	1.130 (0.231)*
My family gamble	1.53 (0.13)*	1.161 (0.237)*
Have gambled on		
Bingo	1.102 (0.133)*	0.402 (0.296)
Pub/club EGMs	0.972 (0.118)*	0.199 (0.269)
Casino (EGMs or tables)	1.514 (0.158)*	0.939 (0.339)*
Internet	1.32 (0.114)*	0.864 (0.256)*
Phone	1.637 (0.18)*	0.548 (0.474)
Satisfied depression criteria	0.496 (0.128)*	0.261 (0.266)
Drinks alcohol on a weekly basis	0.649 (0.078)*	0.246 (0.175)
*Protective factors*		
Connectedness to family	−0.245 (0.052)*	0.068 (0.103)
Connectedness to friends	−0.33 (0.038)*	−0.053 (0.103)
Connectedness to school	−0.412 (0.077)*	−0.326 (0.137)*

* Item/variable significant at *p* ≤ 0.01

^†^Adjusted for age, sex, ethnicity and neighbourhood socio-economic deprivation (NZDep2006)

Model Two entailed a multivariate analysis between the latent gambling continuum, demographic variables, and all of the variables identified in Model One as independently fulfilling risk/protective functions. The analysis in Model Two aimed to determine if those variables identified as fulfilling risk/protective functions (as identified in Model One) would continue to maintain these functions in the presence of each other. The only variable to maintain its protective status was school connectedness (*p* = 0.018). Variables that continued to be significantly associated with increased risk included being of a Pacific ethnicity (*p* = 0.009), earning more than NZ$100 per week (*p* = 0.031), being male (*p* = 0.002), gambling because friends gamble (*p* < 0.001), and gambling because family gambles (*p* < 0.001). Gambling at a casino (on EGMs or tables) and gambling over the Internet were significantly associated with an increased risk of ‘unhealthy gambling’ behaviour (*p* = 0.006 and *p* = 0.001 respectively). Table 2 provides estimates and standard errors for each of these items.

## Discussion

Based on data from a nationally representative sample of secondary school students, the current study has identified a single construct of unhealthy gambling behaviour and a number of ‘red flags’, or indicators, of when gambling behaviour is at or is close to unhealthy levels. For instance, “gambling to relax”, “gambling to feel better about myself”, and “gambling to forget about things” are probable early indicators that gambling behaviour may be at unhealthy levels. It may be useful to incorporate these ‘red flags’ in health promotion and treatment initiatives so that young people and those responsible for their health and wellbeing (e.g. teachers, parents, and clinicians) have an awareness of the warning signs linked to unhealthy gambling behaviour.

The statistical model and ‘red flags’ that were identified in this research resemble a number of aspects of Blaszczynski and Nower’s (2002) second pathway—*emotionally vulnerable problem gamblers,* from their theoretical Pathway Model of problem and pathological gambling conducted with adults. They propose that emotionally vulnerable gamblers are motivated to participate in gambling “by a desire to modulate affective states and/or meet specific psychological needs” (p. 492). Recent research in adolescent problem gambling has further supported Blaszczynski and Nower’s Pathway Model (Gupta et al. 2013), and as in previous research the three ‘red flags’ of unhealthy gambling identified in the present study all appear to centre on reducing states of arousal. Moreover, in our study unhealthy levels of gambling were associated with depression, poorer wellbeing, and weekly use of alcohol. Blaszczynski and Nower (2002) also argue that the underlying psychological dysfunction present in ‘pathway two’ gamblers makes them more resistant to treatment and that underlying vulnerabilities, such as substance misuse, must be addressed alongside gambling issues.

As in the current study, prior work has indicated that those from non-majority ethnic groups (e.g. Native American and African American youth in the USA and indigenous young people in Australia) may not be any more likely to gamble than their ethnic majority peers (Volberg et al. 2010). However, as was observed amongst Pacific young people in the present study, non-majority ethnic youth that do gamble appear to be at greater risk of problem gambling (Delfabbro et al. 2005; Volberg et al. 2010). Previous research has also found that issues with both family and school bonding, or connectedness, are associated with problem gambling (Dickson et al. 2008; Lussier et al. 2007). While this research identified connectedness to friends, family and school as fulfilling protective functions (model one), it is interesting to note that school connectedness was the only domain to maintain this relationship in the presence of other (protective and risk) items (model two). This has important implications for schools in their role in enhancing the overall wellbeing of young people in their possible role towards developing appropriate policies (e.g. policies focused on the availability of gambling and reducing the harm caused by problem gambling amongst adolescents), and their input with targeted interventions aimed at preventing gambling problems.

This research has identified two socio-ecological domains that are important in relation to youth gambling. Firstly, the gambling behaviour of students’ friends and parents were shown to influence students’ gambling. While social learning and/or social attachment theories may assist with the interpretation of this, in-depth qualitative research to fully explore the underlying mechanisms is warranted. Health promotion efforts aimed at education of parents regarding their own acceptance and modelling of gambling behaviour, and the subsequent effects on youth may also be useful. Secondly, this research has highlighted the potency of gambling via modes of gambling typically available at casinos (i.e. EGMs or tables) or the Internet for youth, even when casino gambling for participants in this study was illegal (i.e. casino gambling is only legal in New Zealand for those aged 20 years or older). This corresponds to research that has previously identified a number of attributes thought to contribute to the potency of these modes, including: limited potential for social monitoring; high levels of accessibility (i.e. casinos operate 24 h a day, seven days a week); being continuous in nature (i.e. there is a very short time frame between investment and outcome); universal access for internet gambling (i.e. remotely accessible via smartphones); and, a lack of enforceable age restrictions for internet gambling (Abbott et al. 2014; Abbott and Volberg 2000; Adams et al. 2004; Floros et al. 2013; Health Sponsorship Council 2012; Orford 2011). While it will continue to be difficult to enforce age restrictions for gambling via the Internet, there is a clear need for casinos to be extra vigilant about children and adolescents gaining access to their premises.

Future research could do more to explore the role of protective factors. For example, it would be valuable to analyse the factors and/or conditions associated with maintaining optimal levels of wellness in the young people that have not gambled, and then potentially explore the best ways in which these factors and/or conditions could be enhanced amongst those identified as engaging in problem gambling. More could also be studied in relation to the role of school connectedness, and in particular what it is about a school milieu that protects young people against problem gambling.

### Strengths and limitations

The data presented in this paper are derived from a large nationally representative sample of secondary school students in New Zealand. Questions on gambling were embedded in a holistic survey on overall adolescent health and wellbeing and, as such, gambling can be viewed in a broad socio-ecological context. A limitation of this research is that the sample only included secondary school students. Young people who were disengaged from the education system (i.e. have dropped out of school or were truant) were not surveyed, and this may have implications for the generalisability of the findings, particularly as ‘disengaged’ students are likely to be ‘high-risk’ with regard to problematic gambling behaviour (Messerlian and Derevensky 2005). The survey was carried out in 2007, and since this time certain modes of gambling, in particular Internet gambling, have become more widespread. Finally, the Youth’07 survey is a cross-sectional survey which relies on students self-report. While it provides robust correlational data, causality and directionality of relationships cannot be determined.

## Conclusions

Building upon earlier work, such as Blaszczynski and Nower’s Pathway Model, this research used item response theory to identify a number of ‘red flags’ of unhealthy gambling behaviour in youth. Socio-ecological factors were also highlighted; schools, families and the gambling industries are important in fostering safe engagement by youth with gambling.
